# Supplementary material for: Salinity Stress Alters the Secondary Metabolic Profile of M. sativa, M. arborea and Their Hybrid (Alborea)
Source: Int J Mol Sci. 2021 May 5;22(9):4882. doi: 10.3390/ijms22094882 (PMC8124458; doi:10.3390/ijms22094882)
Supplement: Supplementary file 1 [file ijms-22-04882-s001.zip › ijms-1192079-supplementary.pdf]

Supplementary Materials:

Table S1. VIP Compounds that differentiate the *M. arborea* from the Alborea & *M. sativa* species.

RT [min]	Molecular Weight	Formula	MS/MS	Tentative Identification	Category	Upregulated
7.42	356.0749	$C_{15}H_{16}O_{10}$	147 ($C_9H_7O_2$)	Caffeoyl glucuronide	Hydroxycinnamic acid derivative	Arb
11.17	452.0787	$C_{16}H_{20}O_{15}$	269($C_{16}H_{13}O_4$)	Formononetin	Flavonoid	Arb
12.55	1042.4639	$C_{43}H_{78}O_{28}$	489($C_{30}H_{49}O_5$)	Caryophyllogenin type saponin	Saponin	Arb
12.77	826.4370	$C_{42}H_{66}O_{16}$	489 ($C_{30}H_{49}O_5$)	Caryophyllogenin type saponin	Saponin	Arb
12.89	1220.5465	$C_{57}H_{88}O_{28}$	503 ($C_{30}H_{47}O_6$)	Medicagenic acid saponin	Saponin	Arb
13.03	1088.5048	$C_{52}H_{80}O_{24}$	503 ($C_{30}H_{47}O_6$)	Medicagenic acid saponin	Saponin	Arb
14.94	664.3828	$C_{36}H_{56}O_{11}$	-	Phytolaccasaponin	Saponin	Arb
8.52	486.0460	$C_{22}H_{14}O_{13}$	285 ($C_{18}H_{21}O_3$)	-	Diphenyl	alb,sat
10.88	588.2422	$C_{27}H_{40}O_{14}$	353 ($C_{21}H_{21}O_5$)	Xanthohumol	flavonoid	alb,sat
11.29	828.3437	$C_{39}H_{56}O_{19}$	270 ($C_{15}H_{10}O_5$) 194 ($C_{10}H_{10}O_4$)	Apigenin Ferulic acid	Flavonoid	alb,sat
11.11	1298.6179	$C_{60}H_{98}O_{30}$	518 ($C_{30}H_{46}O_7$)	Cucurbitacin (zahnic acid)	saponin	alb,sat
11.64	1090.5560	$C_{53}H_{86}O_{23}$	-	Medicago-saponin	saponin	alb,sat
11.93	1414.6250	$C_{64}H_{102}O_{34}$	454 $C_{29}H_{42}O_4$	Diosgenin derivative	Flavonoid	alb,sat
13.06	1206.5650	$C_{57}H_{90}O_{27}$	456 ($C_{29}H_{44}O_4$) 393 ($C_{28}H_{40}O$)	O-Acetyldiosgenin+	Flavonoid	Alb, sat
14.37	634.4086	$C_{36}H_{58}O_9$		Ecliptasaponin A		alb,sat
14.77	988.5258	$C_{49}H_{80}O_{20}$	471 ($C_{30}H_{47}O_4$)	Hederagenin derivative Saponin	Saponin	Alb Sat
14.79	780.4659	$C_{42}H_{68}O_{13}$	-	Salikosaponin	Saponin	alb,sat
15.58	704.3773	$C_{38}H_{56}O_{12}$	439 ($C_{29}H_{43}O_3$)	Akebonoic acid type saponin	Saponin	alb,sat
15.41	1028.519	$C_{51}H_{80}O_{21}$	270 ($C_{15}H_{10}O_5$)	Apigenin derivative	Flavonoid	alb,sat
15.7	706.3943	$C_{38}H_{58}O_{12}$	439 ($C_{29}H_{43}O_3$)	Akebonoic acid type saponin	Saponin	Alb Sat

Table S2. Differentiation of *M. sativa* and Alborea species concerning their secondary metabolites content.

Shoots, Alborea vs *M. sativa*.

RT [min]	Molecular Weight	Formula	MS/MS	Tentative Identification	Category	Upregulated
9.88	332.1597	$C_{19}H_{24}O_5$	244 ($C_{18}H_{12}O$)	-	Biphenyl	alb
15.82	386.1727	$C_{22}H_{26}O_6$	-	Eudesmin	Lignan	alb
9.72	682.1382	$C_{29}H_{30}O_{19}$	-	Tenuifoliside B	Phenol	sat
10.69	858.1851	$C_{39}H_{38}O_{22}$	285 ($C_{15}H_9O_6$)	Aureusidin	Flavonoid	sat
10.81	506.1064	$C_{23}H_{22}O_{13}$	286 ($C_{15}H_{10}O_6$)	Luteolin	Flavonoid	sat
13.38	622.1321	$C_{31}H_{26}O_{14}$	270 ($C_{15}H_{10}O_5$)	Apigenin	Flavonoid	sat
13.26	1042.4620	$C_{50}H_{74}O_{23}$	485 ($C_{30}H_{45}O_5$)	Quinovic acid	Saponin	sat

RT [min]	Molecular Weight	Formula	MS/MS	Tentative Identification	Category	Upregulated
11.20	1106.5510	$C_{53}H_{86}O_{24}$	436 ($C_{30}H_{44}O_2$)	Ganoderal	Triterpenoid	sat
13.26	1174.5040	$C_{55}H_{82}O_{27}$	436 ($C_{30}H_{44}O_2$)	Ganoderal	Triterpenoid	sat

Roots, Alborea vs *M. sativa*

RT [min]	Molecular Weight	Formula	MS/MS	Tentative Identification	Category	Upregulated
11.58	254.0579	$C_{15}H_{10}O_4$	-	Daidzein	Isoflavones	alb
15.82	386.1727	$C_{22}H_{26}O_6$	-	Eudesmin	Lignan	alb
12.20	1090.5200	$C_{52}H_{82}O_{24}$	446 ($C_{28}H_{46}O_4$)	Methylspirostane type saponin	Saponin	sat
12.21	1222.5630	$C_{57}H_{90}O_{28}$	446 ($C_{28}H_{46}O_4$)	Methylspirostane type saponin	Saponin	sat
12.21	518.3247	$C_{30}H_{46}O_7$	-	Zanhic acid	Saponin aglycon	sat
15.27	486.3349	$C_{30}H_{46}O_5$	-	Quillaic Acid	Saponin aglycon	sat
15.02	810.4406	$C_{42}H_{66}O_{15}$	-	Esculentoside or Azukisaponin III	Saponin	sat
15.01	824.4197	$C_{42}H_{64}O_{16}$	-	Uralsaponin	Saponin	sat

Table S3. Relative fold changes of differential secondary metabolites for Roots of *M. sativa, M. arborea* and Alborea under salt-stress and salt-sock treatments.

Roots		Log fold change					
		M. arborea		***M. sativa***		**Alborea**	
	Molecular Formula	$Log_2^{(50-75-100/control}$	$Log_2^{(100/control}$	$Log_2^{(50-75-100/control}$	$Log_2^{(100/control}$	$Log_2^{(50-75-100/control}$	$Log_2^{(100/control}$
Saponins	$C_{60}H_{92}O_{28}$	-3.14	ns	ns	ns	ns	ns
	$C_{48}H_{72}O_{19}$	-3.64	ns	ns	ns	ns	ns
	$C_{52}H_{82}O_{24}$	-3.42	ns	ns	ns	ns	ns
	$C_{50}H_{94}O_{33}$	-3.22	ns	ns	ns	ns	ns
	$C_{52}H_{82}O_{24}$	-5.61	ns	ns	ns	ns	ns
	$C_{42}H_{68}O_{15}$	ns	-3.32	ns	ns	ns	ns
	$C_{42}H_{66}O_{15}$	ns	-4.18	ns	ns	ns	ns
	$C_{39}H_{60}O_{13}$	ns	-4.05	ns	ns	ns	ns
	$C_{36}H_{54}O_{12}$	ns	ns	ns	ns	ns	-4,57
	$C_{36}H_{56}O_{11}$	ns	ns	ns	ns	ns	-6,25
	$C_{39}H_{58}O_{14}$	ns	ns	ns	ns	ns	-4.93
	$C_{46}H_{74}O_{17}$	ns	ns	ns	ns	ns	-7,01
	$C_{59}H_{80}O_{17}$	ns	ns	ns	ns	ns	-5,6
Flavonoids	$C_{26}H_{30}O_{12}$	-4.3	ns	ns	ns	ns	ns
	$C_{25}H_{24}O_{13}$	ns	ns	-3,36	ns	ns	ns
	$C_{25}H_{24}O_{12}$	ns	ns	-3,12	ns	ns	ns

	$C_{26}H_{26}O_{13}$	ns	ns	ns	ns	3.41	
Triterpenic acids	$C_{30}H_{46}O_4$	ns	-4.08		ns	ns	ns
	$C_{30}H_{48}O_5$	ns	-3.39		ns	ns	ns
	$C_{30}H_{46}O_6$	ns	ns	ns	ns	ns	-5.33
	$C_{30}H_{46}O_6$	ns	ns	ns	ns	ns	-5.86
	$C_{30}H_{44}O_5$	ns	ns	ns	ns	ns	-5.34
	$C_{30}H_{46}O_6$	ns	ns	ns	ns	ns	-3.84
Lignans	$C_{22}H_{26}O_6$	9.56	ns	6,32	ns	ns	9.4
	$C_{22}H_{26}O_6$	7.32	ns	9,64	ns	ns	ns
	$C_{22}H_{26}O_6$	ns	ns	7.28	ns	ns	ns
Benzyl Tetrahydrofuran		7.50	ns		ns	ns	8.26
	$C_{14}H_{18}O_5$			4,81			
	$C_{14}H_{18}O_5$	7.22	ns	7,29	ns	ns	ns
	$C_{14}H_{18}O_5$			7,34	ns	ns	ns
	$C_{14}H_{18}O_5$			7.39	ns	ns	ns
Phenols	$C_{13}H_{18}O_5$	7.36	ns	7.43	ns	ns	ns
	$C_{33}H_{38}O_6$	ns	ns	-3.71	ns	ns	ns
	$C_{20}H_{22}O_3$	ns	ns	6.56	ns	ns	ns

Table S4. Relative fold changes of differential secondary metabolites for Shoots of *M. sativa, M. arborea* and Alborea under salt-stress and salt-sock treatments.

Shoots		Log fold change					
		M. arborea		***M. sativa***		**Alborea**	
		$Log_2^{(50-75-100/control}$	$Log_2^{(100/control}$	$Log_2^{(50-75-100/control}$	$Log_2^{(100/control}$	$Log_2^{(50-75-100/control}$	$Log_2^{(100/control}$
Flavonoid glycosides	$C_{27}H_{26}O_{17}$	ns	ns	-4.23	ns	ns	ns
	$C_{43}H_{42}O_{26}$	ns	ns	-4.43	ns	ns	ns
	$C_{43}H_{42}O_{26}$	ns	ns	-3.8	ns	ns	ns
	$C_{42}H_{40}O_{25}$	ns	ns	-4.46	ns	ns	ns
	$C_{28}H_{28}O_{18}$	ns	ns	-3.78	ns	ns	ns
	$C_{34}H_{62}O_{22}$	ns	ns	-7.31	ns	ns	ns
Flavonoids	$C_{21}H_{18}O_{11}$	ns	ns		ns	ns	3.23
Triterpenic acids	$C_{30}H_{46}O_6$	ns	ns		ns	ns	3.78
Lignans	$C_{22}H_{26}O_6$	ns	ns	7.65	ns	ns	10.08
	$C_{22}H_{26}O_6$	ns	ns	10.45	ns	ns	7.12
	$C_{20}H_{22}O_3$	ns	ns	7.49	ns	ns	ns
	$C_{22}H_{26}O_6$	ns	ns	8.59	ns	ns	ns
Benzyl tetrahydrofurans							
	$C_{14}H_{18}O_5$	ns	ns		ns	ns	7.47
	$C_{14}H_{18}O_5$	ns	ns		ns	ns	7.5
	$C_{14}H_{18}O_5$	ns	ns		ns	ns	7.57
	$C_{14}H_{18}O_5$	ns	ns	7.87	ns	ns	ns
	$C_{14}H_{18}O_5$	ns	ns	8.22	ns	8.16	ns
	$C_{14}H_{18}O_5$	ns	ns	8.31	ns	ns	ns
	$C_{14}H_{18}O_5$	ns	ns	7.86	ns	ns	ns
Phenols							
	$C_{13}H_{18}O_5$	ns	ns	8.15	ns	ns	ns
	$C_{24}H_{30}O_6$	ns	ns		ns	ns	-4.49

Biphenyls	$C_{24}H_{30}O_6$	ns	ns	-3.24	ns	ns	ns

Table S5. Main secondary metabolites over-expressed in *M. arborea* (green) vs *M. sativa* and Alborea (blue) under the two salinity treatments in roots and shoots

	**Roots**	**Shoots**
**Acute stress (100 mM NaCl)**	Saponins (mainly zahnic and medicagenic acid)<br><br>Lignan (only for Alborea)	Phenolic compounds (Hydroxycinnamic acids)<br><br>Triterpenic saponins flavonoid, lignan (only for Alborea)
**Gradual stress (50-75-100 mM NaCl)**	Saponins<br><br>Flavonoids, phenols, lignan (only for Alborea)	Phenolic compounds (Hydroxycinnamic acids)<br><br>Triterpenic saponins, flavonoid

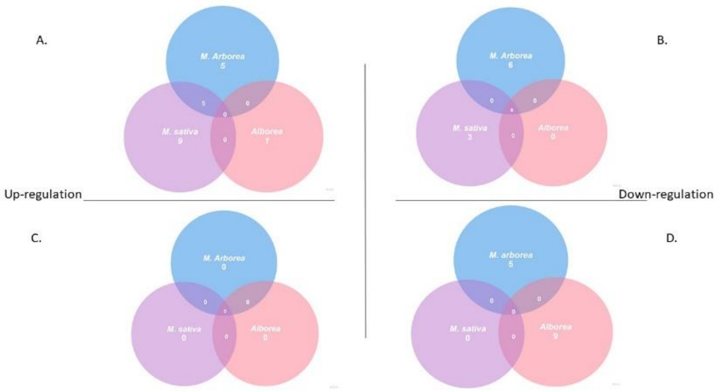

Figure S1. Venn diagrams depicting the total number of metabolites significantly altered (below the entry's name) as well as the overlapping metabolites between the three entries in the roots under the two different salt treatments. A. Up-regulation 50-75-100 mM NaCl, B. down-regulation 50-75-100 mM NaCl, C. Up-regulation 100 mM NaCl, D. Down-regulation 100 mM NaCl

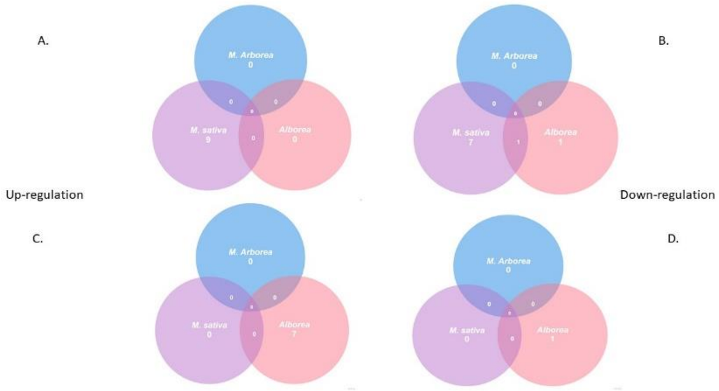

Figure S2. Venn diagrams depicting the total number of metabolites significantly altered (below the entry's name) as well as the overlapping metabolites between the three entries in the shoots under the two different salt treatments. A. Up-regulation 50-75-100 mM NaCl, B. down-regulation 50-75-100 mM NaCl, C. Up-regulation 100 mM NaCl, D. Down-regulation 100 mM NaCl
